# A novel association rule mining approach using TID intermediate itemset

**DOI:** 10.1371/journal.pone.0179703

**Published:** 2018-01-19

**Authors:** Iyad Aqra, Tutut Herawan, Norjihan Abdul Ghani, Adnan Akhunzada, Akhtar Ali, Ramdan Bin Razali, Manzoor Ilahi, Kim-Kwang Raymond Choo

**Affiliations:** 1 Department of Information Systems, University of Malaya, Kuala Lumpur, Malaysia; 2 Department of Computer Science, COMSATS Institute of Information Technology (CIIT), Islamabad, Pakistan; 3 Faculty Electrical Electronics Engineering, University Malaysia Pahang, Pekan, Malaysia; 4 Department of Information Systems and Cyber Security, University of Texas at San Antonio, San Antonio, Texas, United States of America; TNO, NETHERLANDS

## Abstract

Designing an efficient association rule mining (ARM) algorithm for multilevel knowledge-based transactional databases that is appropriate for real-world deployments is of paramount concern. However, dynamic decision making that needs to modify the threshold either to minimize or maximize the output knowledge certainly necessitates the extant state-of-the-art algorithms to rescan the entire database. Subsequently, the process incurs heavy computation cost and is not feasible for real-time applications. The paper addresses efficiently the problem of threshold dynamic updation for a given purpose. The paper contributes by presenting a novel ARM approach that creates an intermediate itemset and applies a threshold to extract categorical frequent itemsets with diverse threshold values. Thus, improving the overall efficiency as we no longer needs to scan the whole database. After the entire itemset is built, we are able to obtain real support without the need of rebuilding the itemset (e.g. Itemset list is intersected to obtain the actual support). Moreover, the algorithm supports to extract many frequent itemsets according to a pre-determined minimum support with an independent purpose. Additionally, the experimental results of our proposed approach demonstrate the capability to be deployed in any mining system in a fully parallel mode; consequently, increasing the efficiency of the real-time association rules discovery process. The proposed approach outperforms the extant state-of-the-art and shows promising results that reduce computation cost, increase accuracy, and produce all possible itemsets.

## Introduction

Association rule mining (ARM) [1] is the most extensively used knowledge discovery technique and a promising field of the mining domain [2–10]. Since the introduction of ARM by Agrawal in [3, 11], ARM has been widely utilized to extract useful and understandable patterns of data from a large amount of data. A challenge associated with ARM is the market basket problem, which has its origins in the study of consumer purchasing patterns in retail stores [12]. However, ARM and data mining have applications beyond this specific setting [13, 14]. The primary aim of extracting knowledge from databases is to generate a large frequent itemset that is iterative. However, it is a complex task because generating frequent itemsets exhausts system resources [2, 5]. For instance, generating candidate itemsets and calculating the occurrence of a candidate set in a transaction set and subsequently in a database involve a number of iterations. Consequently, each iteration requires time and incurs heavy computation cost. Two thresholds are involved, namely; a minimum support, and a minimum confidence[15, 16]. Minimum support function acts as a barrier. For example, if a candidate itemset has an occurrence greater than or equal to it, then the itemset is considered frequent; otherwise, the itemset is avoided. If a rule has confidence greater than the minimum confidence, then it is a strong rule in terms of the knowledge of the output. Generating confidence is utilized to measure the correspondence between two parts of a rule [3, 10]. A number of algorithms have been proposed to manage association rule discovery, such as Apriori [3], Eclat [4], DHP [17], AprioriTID [11], McEclat [5], and MsApriori [16].

The ARM problem can be decomposed into two sub-problems. In the first sub-problem, we need to derive a large itemset that has an occurrence in a database that is greater than the minimum support (minimum support is the input threshold). The second sub-problem is using the output from a previous large frequent itemset to generate an association rule. The first step is more intricate than the second one, and it requires scanning the database multiple times. Finally, it results two main issues. The first issue involves discovering a large frequent itemset based on the input threshold. An item whose occurrence in the database is equal to or more than the minimum support becomes an element in a large frequent itemset. Thus, an element in large frequent itemsets occurs frequently according to fixed minimum support and, it is entered by the user. This condition means that when the decision maker needs to change the threshold to increase or decrease the knowledge volume, the algorithm is forced to rebuild knowledge from the beginning, which consumes resources with heavy computation cost. The second issue involves neither discovering a required rare frequent item without setting up a small minimum support nor using a multilevel minimum support algorithm. However, in the two cases, the large frequent itemset becomes even larger. This condition implies that knowledge extraction needs to be managed. Such management requires another angle to generate a rare itemset. To fully benefit from parallel computing, one would need to modify the Apriori algorithm [5], as the algorithm becomes more efficient when handling vertical layout data. The concept of threshold was proposed to “burn” the candidate itemset that does not exceed minimum support [3–5, 15].

In this study, we present an ARM approach based on Apriori (hereafter referred to as ITDApriori). In our approach, a new itemset format structure is adopted to address the aforementioned issues. The proposed structure achieves high accuracy with an advanced facility. Specifically, a novel alternative perspective is designed to allow extraction with no threshold as a primary parameter and to extract knowledge with minimum support and without scanning the entire database. The contributions of the paper are listed below. Our ITDApriori approach, on the other hand, prepares knowledge or a frequent itemset with all possible itemsets occurring in the database as an intermediate step to obtain the final instance of the frequent itemset. The proposed algorithm also helps to extract many frequent itemsets according to a pre-determined minimum support with an independent purpose. Furthermore, the association rule set is extracted with high confidence and weak support. The main motivation is look for a strong related pattern that is different from other patterns having a rare occurrence. On the contrary, the extant approaches in the data mining field focus on the same main goal, which is to identify the most common frequent pattern in a database. The main contributions of the paper are listed below.

1. The paper presents a novel ARM approach that creates an intermediate itemset and applies a threshold to extract categorical frequent itemsets with diverse threshold values. Thus, improving the overall efficiency as we no longer need the algorithm to rescan the entire database.
2. The algorithm supports to extract many frequent itemsets according to a pre-determined minimum support with an independent purpose.
3. The proposed approach is capable to be deployed in any mining system in a fully parallel mode; consequently, increasing the efficiency of the real-time association rules discovery process and making it feasible for real-time applications.
4. The proposed approach outperforms the extant state-of-the-art and shows promising results that reduce computation cost, increase accuracy, and produce all possible itemsets.

The remainder of this paper is organized as follows. Section 2 presents an overview of ARM. A brief background of the study and a literature review are presented in Section 3. Section 4 describes the intermediate itemset approach. Section 5 presents the evaluations. Section 6 concludes the paper.

## Association rules

Several key terms are utilized in frequent itemset mining and have been specified in the introduction. In this section, we clarify and formulate these expressions to present the fundamental concepts of frequent itemset mining. For a clearer depiction, we employ market basket as an example to exhibit meaning in a significant manner. The following definition describes the notion of item set.

**Definition 1 (Set of item)**: *The item set is defined as I* = {*i*_1_, *i*_2_, *i*_3_, …, *i*_*m*_}, *where i is the item in database transaction*.

It is a chance to be the arrangement of attributes in an item transaction. In other words, for all items in the system, subscript m starts from 1 indicating each item. In the market basket, the item refers to a product in shelves.

From Definition 1, we have the following notion of transaction.

**Definition 2**: *A transaction T is over I is defined as a pair T* = (*tid*, *I′*), *where tid is the transaction identifier and I*′ ⊆ *I*.

At the point when a customer purchases several products, the process will be stored in the database. Two important points need to be noted. The first one is the ID for this process (transaction) (defined as *tid*). The other one is a set of purchased items (defined as *I*′).

From Definition 2, we have the following notion of transaction database.

**Definition 3**: *Transaction database D is a collection of transactions* {*T*} *over I*.

From Definitions 1–3, we have the following notion of itemset.

**Definition 4 (itemset)**: *Let given x* = {*i*_1_, *i*_2_, *i*_3_, ⋯, *i*_*k*_}, *where x itemset*, *it is means the set of items is repeated frequently in a database as a pattern*.

The support of itemset is defined as follow

**Definition 5 (itemset support)**: *The support of itemset x is the number of T in D*

$$Support\;\;\left(x,D\right)=\left|\left\{tid|\left(tid,I^{\prime }\right)\in D,x\subseteq I^{\prime },I\subseteq I^{\prime }\right\}\right|$$

*that contain the itemset x i.e*.

From Definition 5, support represents an itemset in a database transaction; hence, we can consider it the weight of an itemset.

The following example describe how to obtain support of itemset

**Example 1**: If *Z* = {*a*, *b*, *c*} is an itemset, *D* has 10 transactions, and *Z* can be found in 4 transactions, then we have the following support of *Z*

$$Support\left(Z\right)=\frac{4}{10}=0.4.$$

From Definition 5, we have the notion of minimal support threshold

**Definition 6**: *Frequent itemset (Itemset F) is called as a frequent itemset if it has support greater than the specified minimal support threshold σ*, *where* 0 ≤ *σ* ≤ |*D*|. *The collection of frequent itemsets in *Ɗ* with respect to σ is denoted by*

F(D,σ)={x⊆I|Support(x,D)≥σ}

For example, if itemsets *x*_*y*1_, *x*_*y*2_ have support *α*, *β*, respectively, then *α* > *β* means that itemset *x*_*y*1_ is more important than *x*_*y*2_. The itemset *x*_*y*1_ has a greater presence and greater representation in the database transaction than *x*_*y*2_.

From Definition 6, we have the property of frequent itemsets as follow:

**Lemma 1**: (see [18, 19]. *All subsets of frequent itemsets are frequent*.

Mathematically, we suppose that *S* and *T* are sets. If each element of *S* is an element of *T*, set *S* is a subset of the set *T*, and every element in the set *S* has the feature of the elements of the set *T* i.e.

S⊆T⇔∀x∈s⇒x∈T

Lemma 1 is an upshot of the conclusion that is under the meeting of the operation of the set the infrequent and rare. This perception forms the premise of capable pruning methodology based on a research method for frequent itemsets that have been affected by many association mining algorithms. The itemsets were merely observed to be frequent at a past level and should be extended candidates for the current level. The lattice formulation clearly indicates that one need not be restricted to a simple base up the search. The formal notion of an association rules is given as follow.

**Definition 7**: *An association rule is an expression of the form x* ⇒ *y*, *where x*, *y are itemsets and x* ∩ *y* = *ϕ*.

From Definition 7, we have the following notion of association rule support.

**Definition 8**: *The support of an association rule x* ⇒ *y in D is defined as the support of x* ∪ *y i*.*e*.

Support(x⇒y,D)=Support(x∪y,D)

From Definition 8, we have the following notion of association rule confidence.

**Definition 9**: *The confidence of an association rule x* ⇒ *y is the conditional probability of having y contained in a transaction*, *given that x is contained in that transaction*.

$$\text{Confidence}(x\Rightarrow y,D)=\frac{\text{Support}\left(x\cup y,D\right)}{\text{Support}\left(x,D\right)}$$

The following example describes how to obtain the confidence of an association rule.

**Example 2**: Let *D* = {*T*} be a database of transactions. If {*a*, *b*}, {*c*} are itemsets, then the rule {*a*, *b*} = {*c*} in *D* has the following confidence

$$\text{Confidence}=\frac{\text{Support}\;\left(\left\{a,b,c\right\},D\right)}{\text{Support}\;\left(\left\{a,b\right\},D\right)}.$$

The following definition describes the notion of rare items.

**Definition 10**: *The rare itemsets are those items which show up infrequently*, *uncommon in the database*, *that mean it has a low threshold*.

From Definition 10, when this rare itemset covers special cases become more imperative more than frequent itemset. Many researchers consider the rare itemset as a challenge in data mining technique.

Such a rule expresses the association of the transaction, which contains all items in *x*. This transaction also contains all items in *y*. The *x* is called the body or antecedent, and *y* is called the head or consequent of the rule. Moreover, the rule has support and confidence, and both help in the success of minimal support and minimal confidence. In the following section, we present the deep analysis of ARM.

## Background

Although data mining already allows us to generate a good decision, many researchers continue to make it more efficient, professional, and accurate. Researchers have proposed many approaches to deal with knowledge extraction. They have resolved most problems and have responded to the requirements of the work environment as much as possible. Nevertheless, as indicated in section 1, several angles need to be re-evaluated. This section provides a review of previous work related to the current research.

**Fig 1:**
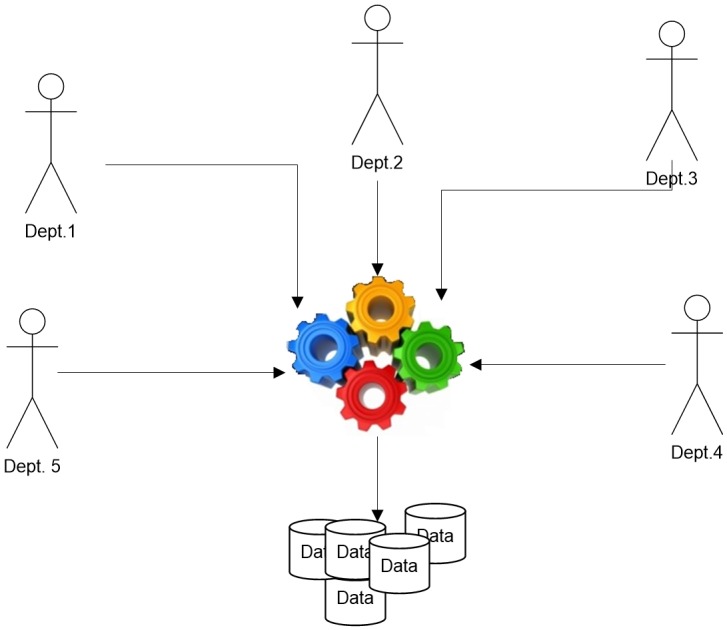
Many users using knowledge.

As shown in Fig 1, when applying an algorithm and obtaining the output knowledge, all users utilize the same extracted knowledge. The knowledge that benefits the arrangement of items on shelves is not meant to arrange the purchase of future deals or to discuss the inability to gain marketing items. We do not really need a method to allow each person or department to review knowledge with a concrete threshold without generating knowledge again from the database. When we introduced association mining in [3], we built it by utilizing the input threshold as a parameter. The threshold has become one of the main parts of association mining generation according to a mining algorithm. This paper discusses several issues to help researchers explore the threshold issue. Separating these issues is difficult because they are interrelated. These issues are discussed in the following paragraph.

There is an assumption, which the size of the itemset is up to 2^*n*^ [4], where n the size of items (things) set in a supermarket. This assumption for discovering an itemset is expected to comprise colossal volumes. Thus, they would be difficult to manage and use. However, an association frequent itemset is an essential step in several data mining techniques, such as associative mining [9, 20, 21], classification mining [11, 20], and clustering mining [22]. These techniques classify the frequent itemset and rule with a suitable means for use either by classifying it as a group or by clustering or by building a classifier to select a suitable rule in association and classification mining. Most of these mining algorithms and methods have been utilized by clients in determining the appropriate minimum support to their databases. This manner alludes to the Apriori-like algorithms [7, 23, 24], which point out that setting the minimum support is entirely unpretentious and can prevent the boundless uses of these algorithms. Our own point of view of the mining transaction database tells us about itemset size that the setting is neither a shape nor a simple form.

Moreover, when an algorithm is produced to discover correlation and frequent patterns in a large database, it was built, in general, over a basket item, as indicated in a popular case study [25], where *I* represents items that exist in the market by a probability of an itemset up to 2^*n*^, where *n* the count of the item in I [21]. According to probability theory, although the above mentioned is true, in a real application, it is impossible to buy all items in the market unless the number of items is small. In a later section, we discuss that is not feasible to buy all items all at once in markets.

As indicated in Lemma 1, burning is a technique that discards the candidate itemset that fails to occur more than the support threshold. The advantages of burning are that it predicts the threshold and reduces the number of times of database scanning. The first algorithm, Apriori, needs to scan a database for each itemset in a candidate set. When several of the candidate itemsets are discarded, the frequency is minuscule. This insinuates that the next candidate set is diminutive; thus, the number of scans with burning is less than that without burning. Several approaches, such as Eclat [11], McEclat [22], and AprioriTID[11], are enhancements of the Apriori algorithm to amass support for the candidate itemset without scanning the database for each candidate itemset. The proposed algorithm needs to scan the database to represent data in a vertical layout in the TID list. The procedure accumulates support through the intersection of the sub itemset TID list in the candidate itemset.

Additionally, burning a handle to avoid worrying about the size of the frequent itemset makes the size diminutive, which is based on the threshold used by users. But, by burning we lose the rare itemset, which is necessary for several applications such as the web and real-time applications. Overall, the computational expense is low in consideration of the promising results obtained for the quality measures. In other words, the largest amount of time is spent generating the itemset, collecting support for the itemset, and checking whether the itemset is related to the threshold. In the following sub-section, we present the deep analysis of Apriori.

### Apriori

Depending on *D*, *I*, *T*, *x*, we can apply association rule mining according to [3]. The authors generally presented and defined the ARM problem. The itemset pattern that represents the entire data in the database is identified. This pattern is called the frequent itemset. Afterward, an association rule is established to detect the correlation between frequent itemsets. The Apriori algorithm is generally one of the most popular and important approaches in data mining. It has become a common frequent itemset pattern technique, similar to association rule mining.

The ARM Process can be decomposed into two main sub-processes. The first one involves finding all *x*, *y* itemsets with *Support* greater minsupp; the general execution of ARM is controlled by the first step, so it is likely to increase performance. The second step is to generate a strong rule. After large frequent itemsets are recognized, the corresponding association rules are determined in a direct manner. Minsupp and minconf are called the threshold, the selection of which poses a popular problem in ARM. Example 3 has demonstrated the Apriori algorithm.

**Example 3**: We consider database D consisting of 10 transactions (See Table 1). We suppose that the minimal support count required is 3 (30%) and let the required minimum confidence be 70%.

**Table 1:** Sample transaction.

TID	Items
1	a,b,e
2	b,d
3	b,c
4	a,b,d
5	a,c
6	b,c
7	a,c,e
8	a,b,c
9	a,b,c
10	a,c

1. The frequent itemset is determined by using the Apriori algorithm.
2. An association rule is generated by using minimum support and minimum confidence.

The first step is to set all items as candidate itemset *C*_1_ = {{*a*}, {*b*}, {*c*}, {*d*}, {*e*}} and scan the database to collect actual support for each item, as shown in Table 2.

**Table 2:** C1, first candidate.

Itemset	Support
{a}	7
{b}	7
{c}	7
{d}	2
{e}	2

When minimum support is applied to candidate set *C*_1_, we obtain *F*_1_ = {{*a*}, {*b*}, {*c*}} in Table 3 as the first frequent itemsets. The pruning step helps to avoid heavy computation caused by a large succeeding candidate set as Apriori property. Two items {{*d*}, {*e*}}, exist in C1 (Table 2). Both items are discarded, from F1, as they do not occur in a transaction up to the minimum support threshold.

**Table 3:** F1, first frequent.

Itemset	Support
{a}	7
{b}	7
{c}	7

The second iteration of the algorithm produces *C*_2_ by using *F*_1_ together with *F*_1_*C*_2_ = *F*_1_|×|*F*_1_. *C*_2_ = {{*a*, *b*},{*a*, *c*},{*b*, *c*}} in Table 4.

**Table 4:** C2, second candidate.

Itemset	Support
{a,b}	4
{a,c}	5
{b,c}	4

When we scanned the database to find a repetition of *C*_2_, we found that all items achieve the minimum support threshold. The *F*_2_ frequent itemset in Table 5 is similar to the *C*_2_ candidate itemset in Table 4.

**Table 5:** F2, second frequent.

Itemset	Support
{a,b}	4
{a,c}	5
{b,c}	4

In the next iteration, the generation of the set of three candidate itemsets *C*_3_ involves the use of the Apriori property. To find *C*_3_, we compute *F*_2_ together with *F*_2_. *C*_3_ = *F*_2_|×|*F*_2_ = {{*a*, *b*, *c*}} (Table 6). Afterward, the “prune” step is employed to reduce the size of *C*_3_. The prune property discards *C*_3_ because it does not help to the success of the support threshold.

**Table 6:** C3 candidate itemset.

Itemset	Support
{a,b,c}	2

Apparently, the Apriori algorithm has good properties, the most popular of which is pruning. Meanwhile, many rare itemsets exist, which will be discarded from the frequent itemset. There is a good enhancement for Apriori to deal with rare Itemset, it is MS-Apriori. The explanation is given in next sub-section over MS-Apriori.

### Multiple-support apriori(MS-Apriori)

The Apriori algorithm can well extract the frequent itemset. However, it has several limitations, such as threshold minimum support and multi-scan database. Hence, many researchers dealt with the concept of the threshold to enhance the algorithm. Most of the presented algorithms in ARM work with single minimum support. The items have a high occurrence in a large frequent itemset, and rare items can be removed from a frequent itemset. If the minimum support is small, the frequent itemset is large, and many useless rules are generated. Given that an enhancement approach for the discovery of frequent itemsets involves uncommon or rare items, multiple-support Apriori (MS-Apriori) was presented in [26]. In this approach, the user can assign a minimum support value for each item. This value is known as the minimum item support (MIS). Frequent itemsets are extracted based on it. By using MS-Apriori, the discovery of frequent itemsets satisfies the lowest MIS value among the items in the itemset. In Example 4, we demonstrate the MS-Apriori algorithm. The MS-Apriori algorithm is not the only algorithm working on the threshold issue. Many researchers are doing work on thresholds [7, 15, 16, 23, 24, 26–28]. This situation implies the value of minimum support, which is large. Minimum support also has high importance in the data mining research environment. The clarification, for Ms-Apriori, has been demonstrated in Example 4.

**Example 4**: We assume the existence of the 10 transactions shown in Table 7. These transactions are utilized for mining. Each transaction is composed of two feature components: transaction identification (TID) and items bought. The predefined minimum support values for items are characterized in Table 8. The goal of this example is to discover the frequent itemset from the data in Table 7 with the multiple predefined minimum support values. The proposed mining algorithm proceeds as follows.

**Table 7:** Dataset, transaction database.

TID	Items
1	ABDG
2	BDE
3	ABCEF
4	BDEG
5	ABCEF
6	BEG
7	ACDE
8	BE
9	ABEF
10	ACDE

**Table 8:** The predefined minimum support values for items.

Item	A	B	C	D	E	F	G
**MIS**	0.4	0.7	0.3	0.7	0.6	0.2	0.4

The first task in ARM is to find the first frequent itemset. In this manner, the tally count and support of each item are obtained. These transactions are shown in Table 7, and item A can be regarded as a sample. The count of item A is 6, and its support value is computed as 6/10 = 0.6. The support estimation values that consider all the items for the 10 transactions are indicated in Table 9. The support value of each item is compared with its predefined minimum support value. Given that the support values of items A, B, C, E, and F are larger than or equal to their predefined minimum supports, these five items are placed in the large single-itemset *F*_1_.

**Table 9:** The support values of all the items for the given 10 transactions.

Item	A	B	C	D	E	F	G
**Support**	0.6	0.8	0.4	0.5	0.9	0.3	0.3

After obtaining *F*_1_, candidate itemset *C*_2_ is produced from *F*_1_, and the support values of the two items (two-itemset) in each itemset in *C*_2_ must be larger than or equivalent to the maximum of their predefined minimum support values. The conceivable candidate two-itemset {A, C} can be regarded as a sample. The support of items A and C are 0.6 and 0.4 from *F*_1_, and the maximum of their minimum support values is 0.4. Considering that both of the supports of these two items are larger than 0.4, the itemset {A, C} is placed in the arrangement set of candidate two-itemset. Having another possible candidate two-itemset {A, B} is excluded. Given that the support (0.6) of item A is smaller than the maximum (0.7) of its minimum support values, itemset {A, B} will be excluded from *C*_2_. All the candidate two-itemsets generated along these lines are determined as *C*_2_ = {{A, C}, {A, E}, {B, E}, {C, F}}. The count and support of each candidate itemset in *C*_2_ is obtained from the given transactions. The calculations are shown in Table 10.

**Table 10:** The support values of all the candidate 2-itemsets.

Item	A, C	A, E	B, E	C, F
**Support**	0.4	0.5	0.7	0.2

The support value of each candidate two-itemset is then compared with the maximum of the minimum support values of the items contained in the itemset. Given that the support values of all the candidate two-itemsets {A, C} and {B, E} fulfill the above condition, these two itemsets are then placed in the set of large two-itemset *F*_2_. After *F*_n_ iterations, we check if *F*_n_ is not null; then, *C*_*n*+1_ is produced. If *F*_2_ is not null, *C*_3_ is produced. The probabilities for *C*_3_ are {{A, B, C}, {A, C, E}, {A, B, E}, {B, C, E}}, but no one of them satisfy the conditions. Where the first one will be excluded because {*B*, *C*} ∉ *F*_2_. For {C, E}, {A, B}, {A, E}, and {B, C, E}, the subsets {C, E} and {B, C} are not frequent in *F*_2_. Hence, we must exclude {B, C, E} from *C*_3_. After that *C*_3_ becomes empty *C*_3_ ⊆ *ϕ*. This condition means that candidate three-itemset C3 does not exist. Similarly, *F*_3_ does not exist. In other words, if *F*_3_ is null, we can discover all frequent itemsets. After collecting the frequent itemsets, the next step is to generate the association rule. Briefly, other related work in ARM Eclat has been described in the next sub-section.

### Eclat and McEclat

Most transaction databases have a horizontal database format. In [4, 5], the proposed method depends on an internal representing database, which is a shift from horizontal format to vertical format (TID). The Eclat algorithm is utilized to conduct itemset mining. It employs the intersection between transaction ID list and TID list to compute the support of a candidate itemset. This work is an improvement because it reduces the number of times a database is scanned. Hence, mining efficiency is increased. In the proposed algorithm, a database is covered to only represent transactions in a vertical format in a TID list. By collecting the TID for each item in item single-itemset, the item’s occurrence in the TID list is collected. After this support is found for K+1-itemset, the intersection is only made between TID lists in the K-itemset. In the following sub-section, the review of ARMGA Algorithm and his enhancements will be coved.

### ARMGA algorithm

Innovatively, a group of researchers [23] presented the hybrid association rule mining with genetic algorithm (ARMGA) algorithm. The creators utilized biogenetic approaches to create a genetic algorithm that deals with the association mining problem without a threshold. In the presented algorithm, users are not required to provide input parameters as a threshold for the algorithm. The selection of an itemset in the proposed algorithm is called ARMGA. The philosophy of the algorithm involves extracting a strong rule with high confidence and without any pre-value for the confidence threshold. The algorithm looks for a rule that has confidence greater than the support of the consequent part of the rule’s confidence (*x* ⇒ *y*) ≥ support (*y*). This algorithm can well implement association rule mining, but the authors attempted to reproduce the original burn infrequent itemset introduced by the father of ARM in the Apriori algorithm. According to the original definition of the association rule, two steps are involved in the discovery of the association rule. The first step is to extract a frequent pattern from a database called a frequent itemset. The second step is the derivation of the rule set from an extracted pattern. In other words, the problem of association rule discovery is decomposed into two sub-problems. The first problem is the search for a common pattern, and the other is the derivation of the rule set from a common pattern. In most cases, the first step is implemented by another data mining technique, such as classification or clustering while the discovery correlation common itemset pattern is a fundamental step in many mining techniques. In this approach, the authors combined two steps of the process: looking for patterns and generating a rule. Many side effects were encountered. Some of them were good, but the others complicated the process. One of the good effects of this approach is that it is efficient for supervised learning or classification learning when the data tuple has a class. As an exclusive consequence, the rule one is looking for is generated. Meanwhile, discovering the frequent pattern in itself is the main part of many data mining techniques. In addition, many of these databases do not have classes. The association rule, by definition, is *x* → *y*, where both *x*, *y* are frequent, meaning that *y* contains an itemset whose length is from 1 and above. When the length of *y* is 1, for example, the rule consequence is a class, meaning that this approach is effective. Otherwise, more difficulties will arise. In the same direction as that of the above-described algorithm, the proposed algorithm in [29] is called G3PARM. It uses the same technique as the algorithm called genetic methodology. The researchers in [29] discussed the objectives of the proposed approach. These objectives are as follows: (1) to reduce gaps in quantitative association rules, (2) to employ fitness patterns, and (3) to avoid misleading interval rules. The minor common property between the proposed algorithm and ITDApriori is to diminish the number of parameters, with a specific end goal of advancing association rule discovery algorithms and gathering an incredible advantage for typical clients.

The main goal of the G3PARM algorithm (and ARMGA as the enhanced algorithm) is to limit and reduce the threshold parameters. However, another calculation is made at the same time to increase the accuracy of the discovery association rule, which would, in turn, increase the possible time of the process of discovering the pattern. The authors did their best in the proposed approach; they worked to reduce many gaps. With regard to the methodology, the researchers were creative in dealing with the discretization of continuous numeric attributes to make them discrete or to categorize the numeric attributes, which is a good contribution to the data mining area. The minor gap in the threshold problem remains. Therefore, the aim of the algorithm presented in the current study is to enhance the previous algorithms. In the following section, we present our proposed approach.

## Proposed approach

This section presents an incipient novelty which is called an intermediate TID Apriori (ITD Apriori). We employ a new data structure that improves the itemset to deal with threshold issues. The developed intermediate TID will help users discover all possible patterns in the database. When all patterns have been collected, the clients can derive many frequent itemsets and rule sets according to a certain threshold without the need to rescan the database. In the 4.1 sub-section, the itemset data structure has been discussed.

### Intermediate transaction ID itemset (ITDM) list data structure

Our intermediate itemset has a new structure it called intermediate transaction Id itemset ITDM list; this structure will increase the efficiency of mining. This can be done by scanning the database and by representing data to a vertical data format. After this process, support can be collected by the intersection TID list. The following definition captured the ITDM data structure.

**Definition 11**: *ITDM list is defined as set*

M={itemset,Support,{tid}}

*Where itemset is a subset from I*, *the support is the support for itemset*, *and* {*tid*} *the transactions ID list that contains itemset*.

The M is an element in ITDM list, the internal part of *M* accessed by *M*.*itemset*, *M*.*Support*, *M*.*tid*. From Definition 11, itemset *F* will be called a frequent itemset if it has *support* greater than the specified minimal support threshold *σ*, where 0 ≤ *σ* ≤ |D|. The collection of frequent itemsets in *Ɗ* with respect to *σ* is defined as

F(D,σ)={x⊆I|Support(x,D)≥σ}.

Frequent itemset mining is related to discovering the set of itemsets F. Items can be any kind of attribute-value pairs; thus, they can also represent the absence of an item *i*_*2*_ in the presence of another item *i*_*1*._

As shown in Table 11, the intermediate itemset has three parts. The first part is set of an itemset *I* = {*x*, *y*, *z*, *w*}, where each is an itemset. The second part {7,6,5,3}, represents the actual support for the itemset, which is a count of TID. The *m*_*i*_.*tid* intersection is used to collect the transactions that contain the itemset *m*_*i*+1_, which makes the auxiliary data structure ready for users to generate knowledge based on the data. In the following sub-section, we present the Idea of generation the set of ITDM, and describe how it is useful.

**Table 11:** ITDM list example.

Itemset	Support	TID List {Transaction ID}						
**X**	7	T1	T2	T3	T4	T6	T7	T8
**Y**	6	T1	T2	T4	T5	T6	T8	
**Z**	5	T1	T3	T4	T6	T7		
**W**	3	T1	T4	T5				

### Generate ITDM set

Most researchers have shown that the most difficult step in association mining is to find a frequent itemset. The discovery of a frequent itemset includes a sub non-trivial iterative process. The two most complex steps are (1) to combine two sub-itemsets to generate a new candidate set and (2) to scan the database to collect support for the new candidate itemset. The second step is solved by the Eclat approach that utilizes the intersection between TIDs. The first one is more complicated based on an approach that only accepts the generation of the candidate set *C*_*i*_ from *F*_*xi*−1_, *F*_*yi*−1_, where *F*_*xi*−1_, *F*_*yi*−1_ are two itemsets in the *F*_*i*−1_ frequent itemset. As a result, a new problem with wise levels arises [9]. These levels are parallel, but parallelism cannot be applied to all candidate sets at one time. To achieve this, we suggest generating candidate set for one time as a template. Afterward, we can collect support within two steps. The first step is to collect actual support for the itemset size one (for all individual items). The second step is to make an intersection to collect support for all parallel itemsets at once. The following example describes how to generate ITDM list. The Example 5 clarify the idea of ITDM.

**Example 5**: The example below demonstrates an ITD Apriori perspective, and it captures generating ITDM list. For a case in point, we have itemset *I* = {*a*, *b*, *c*}. Table 12 contains five transactions of the transactional database. In a normal transaction, the first column is for TID, and the second is for the purchased itemsets in each transaction.

**Table 12:** Database transaction sample.

TID	Items
1	a,b
2	a,c
3	b,c
4	a,c
5	a,b,c

Given that the data structure is shown for the intermediate set, Table 13 presents the first ITDM list that contains only one item in itemset part. The first column is the itemset set, the second column is actual support, and the third column is the list of transactions that contain the itemset. This is the first ITDM *M*_1_. In other words, the database layout is converted from a horizontal format (Table 11) to a vertical format (Table 13), which contains only one itemset.

**Table 13:** First ITDM list.

Itemset	Support	TID
A	4	1, 2, 4, 5
B	3	1, 3,5
C	4	2, 3, 4, 5

The possibility of the candidate itemset is {*ab*, *bc*, *ac*, *abc*}, as shown in Table 14. After collecting the first itemset, we can collect actual support for itemsets in Table 14 for all candidates. This can be done by the parallel intersection of the first itemset (referred to as reinforcement parallel).

**Table 14:** All ITDM list.

Itemset	Support	TID
ab	2	1, 5
ac	3	2, 4,5
bc	2	3, 5
abc	1	5

**Fig 2:**
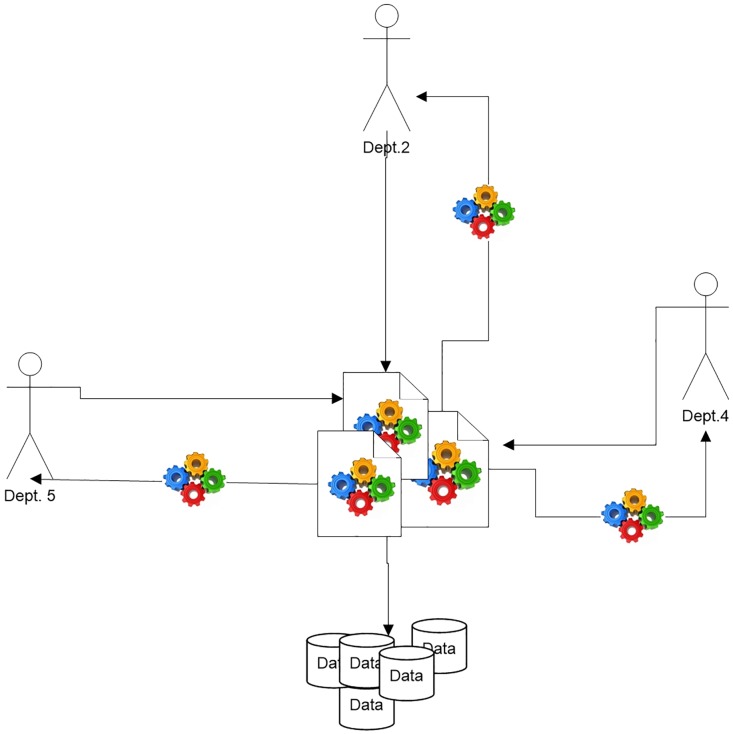
Multi knowledge (Frequent itemset) from ITDM list.

When ITD Apriori collects the ITDM list from a large database, many users can apply a multi-support threshold depending on what the user requires as shown in the drawing symbol in Fig 2. Afterward, each user can extract specific frequent itemsets and rule knowledge when needed. Generation of the candidate set and frequent itemset in the existing approach is limited in terms of composing two frequent itemsets from *F*_*i*−1_; thus, one candidate set *C*_*i*_ is produced. ITDApriori involves three steps as follows.

1. Collecting the first ITDM list.
2. Generating all candidate itemsets up to *C*_*i*_ and formalizing them as an ITDM structure (Table 11, Definition 11), where *I* = maximum count of items in the transaction. By generating all itemsets up to *I*, we generate all itemsets having been represented in the database. In the next section, we discuss and ensure that the itemset contains a full representation for the database. This generation will be only once because there is no change in the itemset. If a new item is added, deleted, or frozen, a new ITDM list will be generated.
3. After generating all candidates and collecting the first item, we can collect support for the candidate itemset, which is parallel to the intersection of the first itemset.

The explanations, over the ITD Apriori, has detailed in the coming sub-section.

### Algorithm (ITD apriori)

**Fig 3:**
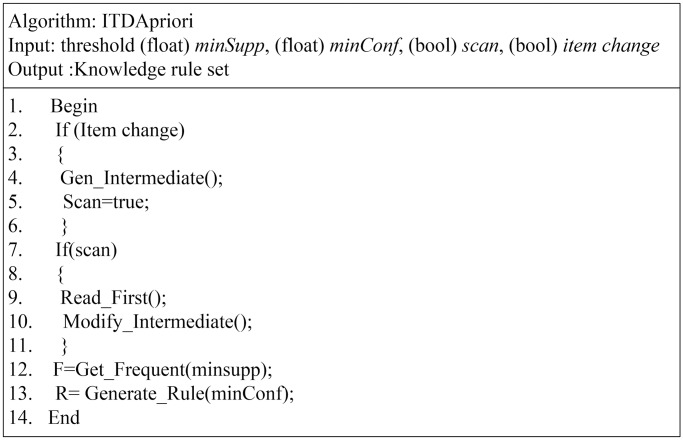
Proposed ITDApriori algorithm.

ITD Apriori presents a new data structure. Small changes need to be implemented in the existing algorithm. In the proposed algorithm, many inputs affect the process and results; these inputs include *scan* and *item change*, both of which are a Boolean data type. The *Scan* is to determine if the user needs to collect from a recent database to change or to derive a new frequent itemset and only a rule set. *Item change* checks the set of a new item added, deleted, or frozen. If there are changes to build a new ITDM list, we can modify the item change parameter in two ways. The first means is manual, where the user can enter the parameter value immediately when the user needs to check whether there are changes in the original itemset. The second means is a sensor status in which some changes exist in the itemset; this would be during a period between the last times of building an ITDM up to the time when some changes occurred in the itemset. These changes will be detected automatically, so the set value is true. When the value of item change is true, the ITD Apriori goes on to build an ITDM from the beginning; otherwise, the algorithm collects the support only without building a candidate set. This would increase the effectiveness of the data mining process when most resources are consumed by the generation of the candidate set, pruning of the itemset, and collection of actual support. In other words, as we aim for the ITDM to extract many instances of knowledge that were already extracted from the database, this process (take an instance from ITDM) occurs many times over the ITDM. The ITDM is extracted once only, and this process will be implemented without needing to rescan the databases again. The user can collect the changes from the database if the flag *scan* is true. In this case, the actual support for each itemset in the ITDM can be collected. This can be done by scanning the database, which will help in the collection of the actual support for the itemset. The pseudo code of the proposed algorithm for generating the ITDM is shown in Fig 3.

The Fig 3 also shows the abstract form of the algorithm. The algorithm in Fig 3 shows the input argument parameters: threshold support, confidence, and scan. In conditional instruction, in line five, the flag of item change in the original itemset is checked. If the flag item change is true, the algorithm will execute the two instructions between brackets, which is Gen_Intermediate method and (scan = true). For this portion, the following scenarios may exist.

1. The ITDM has not been built yet, so the flag must be true. The default value will remain true until the first ITDM is built. After that, the item will be changed to false.
2. The ITDM has already been built.

The main difference between Gen_Intermediate and other algorithms, especial those build over Apriori approach, is Gen_Intermediate build the itemset one time only and in next mining just update support for the element of itemsets. Another difference is no burning strategy exists in the enhancement and length of the itemset up to k, where k is the maximum count item in the database row.

As shown in Table 15, there are two important parameters in proposed algorithm: item change, scan. Where item change represents the stability of item set, if the user modifies the item set this parameter will change to true, and if it changes to true immediately the scan parameter it will has true value, in this case, the algorithm rebuild ITDM from the beginning. Also, as a show, it is not applicable So, as in line 9 from the algorithm, if the flag scan is true, then the database must be scanned and the updated database must be collected, and the user can change the parameter scan to true in order to modify the existing ITDM. Moreover, the value of item change is related to the state of the item set in the system if there are any changes in the item set the value of this parameter must be true, otherwise the user, also, can set it to true in order to rebuild the whole ITDM from the beginning. However, generating the candidate itemset in the proposed enhancement for the algorithm is close to the original algorithm. The critical change in the algorithm is the fact that no burning strategy is applied to a new candidate itemset.

**Table 15:** Item change and scan probability and it effects.

Parameter	Action \ Parameter	Item change = true	Item change = false
Scan = true	Build itemset	✓	✘
	Update itemset	✓	✓
Scan = false	Build itemset	Un Applicable	✘
	Update itemset		✘

**Fig 4:**
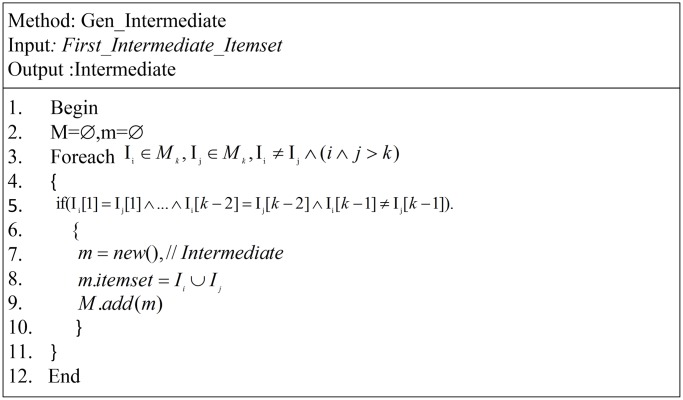
Generate intermediate set method.

The generation of an ITDM abstract level procedure is described in Fig 4. This procedure is the main part of our approach. This procedure combines items to generate an entire ITDM. This procedure is slightly similar to the Apriori generation of the candidate itemset. The main difference is that the Apriori algorithm adopts the burn strategy to reduce the itemset size. In our approach, no burning technique is applied. Initially, the Apriori algorithm appears to be better because it reduces the size of the itemset and discards the infrequent itemset. Moreover, the Existing methods are only discovering one instance of the frequent itemset according to a specific threshold. If there is a change in certain criteria, the algorithms will rescan the database. In the current approach, the algorithm does not need to rescan the database even when the criteria change. Another main difference is that it is not necessary to rebuild an ITDM list in the future. All we need is to build it for the first time or if a change occurs in the original itemset (add or delete items from the system).

Line two shows us two variables M, m. the first one is list contain the large itemset, and from this list the instances of itemset are going to be derived from it. The other one, m, is just one element of the ITDM. We are able to see in line 7 to 9 how the algorithm initials the m object and assign the m.itemset value.

$$foreach{\;\text{I}}_{\text{i}}\in M_{k},{\;\text{I}}_{\text{j}}\in M_{k},{\text{I}}_{\text{i}}\neq {\text{I}}_{\text{j}}\wedge (i\wedge j>k),$$

The above statement, in line 6, is to be read as two itemsets *I*_*i*_, *I*_*j*_ to combine both of them. And both of them are belong to *M*_*k*_ itemsets, and i, j, and k the size of the itemset. This part of the algorithm is the most difficult and is the core part of the discovery pattern in the database, even in the Apriori algorithm; hence, it has high importance. The most important problem in this section of the algorithm is that level wise, it consumes time waiting to combine the next level. To address this issue, the ITDApriori will execute this process one time only until the client makes a change in the original itemset. If the algorithm is applied to look for relation in a database attribute, we need to build the ITDM one time.

**Fig 5:**
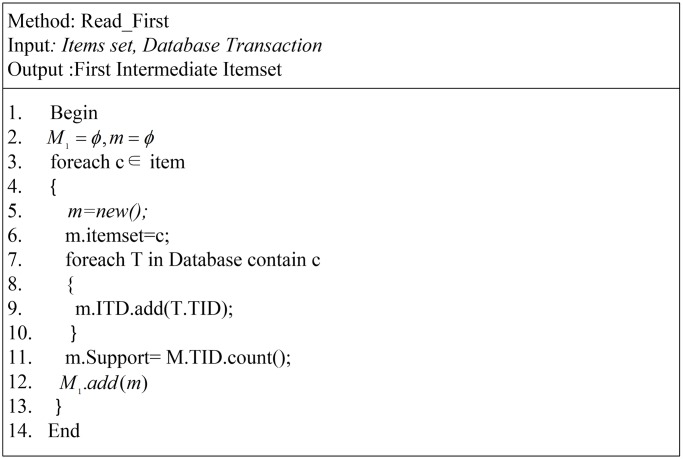
Read first intermediate itemset.

In Fig 5, the function of generating the first ITDM is shown in a pseudo-code form. The aim of this procedure is to collect the ITDM with the ITDApriori structure *m* = {*c*, *Support*, <*TID*>}, and collect actual support for the first itemset from the database. In other words; this procedure is merely a preparatory stage for other coming stages. This idea prepares data for the data mining process, as stated by many researchers and originally stated in this article [30]. This method was not only stated by Fayyad in [30], but is also a creative method presented in many data mining approaches that convert data layout from horizontal to vertical, as presented in [4, 5]. This method is important because in the next iteration, the algorithm needs to perform an intersection on the *m*.*TID* list to collect actual support for the generated itemset.

**Fig 6:**
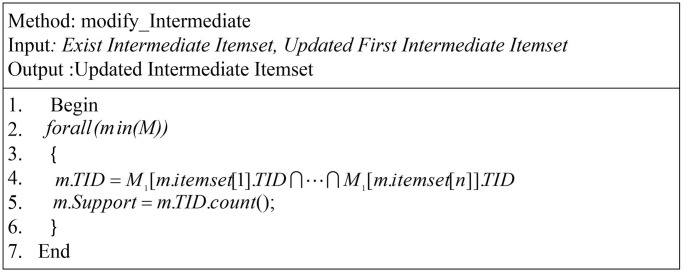
Modify intermediate itemset.

In Fig 6, we show the most important method of ITDApriori. We do not need to create an entire intermediate itemset. We mined the database until some change occurred in the original itemsets in the system. This is the most important component of the proposed approach. Meanwhile, this component cannot be observed in other algorithms, most of which need to create an entire itemset through an iterative process, which devours resources. Hence, next time, the algorithm needs only to read the first itemset and modify the entire itemset by making an intersection in the first itemset to collect new actual support.

In line four in Fig 6, this instruction to form intersection for TID list for item belongs to m itemset. Where M is complete ITDM, m is instance from M, and *M*_1_ is first ITDM. For example if *m*.*itemset* = {*a*, *b*, *d*}, this instruction go to TID for a,b,d and make intersection gather TID for m itemset. Moreover, the modify_Intermediate method has high importance, when the ITDM designed that mean we need to collect the first itemset TID list, after that all itemset TID collected in parallel.

**Fig 7:**
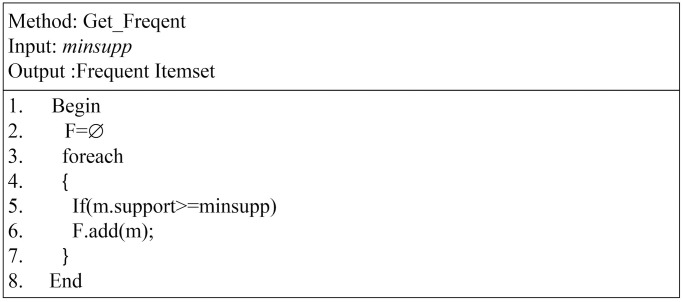
Get frequent itemset.

As shown in Fig 7, many approaches still need to apply a threshold. The proposed approach has no problem with applying a threshold. One can select from two scenarios. The first is to deal with the entire itemset set without a threshold, and the second one is to extract a subset itemset according to a certain threshold. Thus, one can apply a specific threshold either by minimal support or by minimal confidence. The feature of obtaining a subset pattern according to the support threshold in *Get*_*Frequent* the method is available to any user. To extract a frequent itemset pattern, we apply minsupp threshold on the ITD. In the proposed technique, the frequent item is isolated without scanning the database and without modifying the ITDM to allow another exact frequent itemset. The last instruction in the main method is to generate the rule set, similar to Apriori.

The explanation, for ITD Apriori, has been evinced in Example 6.

**Example 6**: Table 16 shows a set of transactions to conduct the ITDM. The original database must be scanned to generate the first ITDM at one time. Afterward, we make convergence in the first list to collect the TID list for all ITDM. The database transactions need to be fully scanned to collect the TID for each item. The following example shows the solution represented in the procedures and steps for obtaining the ITDM.

**Table 16:** Sample of database transaction.

ID	Transactions
T1	a b
T2	b d
T3	b c
T4	a b d
T5	a c
T6	b c
T7	a c
T8	a b c
T9	a b c
T10	b d

A full ITDM can be obtained in many steps. The first step is to collect the first itemset through full scanning. The first itemset is shown in Table 17. The actual support for each item and the list of TID where the item accrues in are also shown.

**Table 17:** First intermediate itemset.

Itemset	Support	TID
a	6	T1,T4,T5,T7,T8,T9
b	7	T1,T2,T3,T4,T6,T8,T9
c	6	T3,T5,T6,T7,T8,T9
d	3	T2,T4,T10

The second step is as follows: if candidate n itemset has already been created from the last mining instance, an intersection between the first itemset only needs to be made. However, if this situation does not exist, n candidate set must be created for all itemsets, as shown in Table 18.

**Table 18:** Second intermediate itemset.

Itemset	Support	TID
ab	4	T1,T4,T8,T9
ac	4	T5,T7,T8,T9
ad	0	
bc	3	T6,T8,T9
bd	2	T2,T4
cd	0	

This process is similar to that in the Apriori algorithm. After generating the itemset set and making an intersection, all the itemsets with actual support are obtained, as shown in Table 19.

**Table 19:** Third intermediate itemset.

Itemset	Support	TID
abc	2	T8,T9
abd	0	0
acd	0	0
bcd	0	0

After completing the ITDM that appears in Table 20, a particular regular frequent itemset and a rule set can be obtained by inserting a threshold. For example, if three pieces of knowledge with support thresholds of 9%, 12%, and 21% are to be extracted, the one with 9%, which is a complete ITDM, is selected. In this case, all itemsets have actual support greater than 9%. The second threshold is 12%. All items that have actual support greater than 12% occur more than once in the transaction set in the database. The frequent itemset is shown in Table 21.

**Table 20:** Final intermediate itemset.

Itemset	Support	TID
ab	4	T1,T4,T8,T9
ac	4	T5,T7,T8,T9
bc	3	T6,T8,T9
bd	2	T2,T4
abc	2	T8,T9

**Table 21:** Frequent itemset with (12% minsupp)—Example2.

Itemset	Support	TID
ab	4	T1,T4,T8,T9
ac	4	T5,T7,T8,T9
bc	3	T6,T8,T9
bd	2	T2,T4
abc	2	T8,T9

From Table 21, the third threshold is 21%. All items have actual support greater than 21%. The frequent itemset is shown in Table 22.

**Table 22:** Frequent itemset with (21% minsupp).

Itemset	Support	TID
ab	4	T1,T4,T8,T9
ac	4	T5,T7,T8,T9
bc	3	T6,T8,T9

We can extricate three instances of frequent itemsets from the intermediate itemset without needing to rescan the database. This saves time and resources and increases the proficiency of mining. By utilizing the ITDM, we implemented the approach, extracted the itemset without a threshold, and embedded the approaches that generate frequent item sets with a threshold. In the next section, the result of ITD Apriori has been presented and discussed.

## Experiment results

Most current algorithms were built over worrying regarding the scale of the frequent itemset, the size of the rule set, and the work that continues to be tiny with the high representative database, according to probably rule, the size of the itemset is up to 2^*n*^ where n is the number of feature or item in the market. This worrying establishes a vital rule: we need to rescan the database on the off-chance that we have to change the threshold in the existing algorithm. This section has four sub-sections the first one is to mention for the dataset that used in the experiments. The second sub-section is for statistical study. The third one is for scalability of the ITD Apriori. The last sub-section shows the comparison of the ITD Apriori with the current state-of-the-art algorithms.

### Dataset

In this section we describe the data sets used in experiment

1. Apriori Dataset: It has 75,000 market basket transactions, it has set of item 49 items, and it represents market basket. It is branch mark dataset. It is has generated by (trac Integrated & Project Management). The Apriori dataset has used in comparison experiments. And there is three four size of this dataset, they have used in scale experiment. The size, of this dataset, on disk is 2.70MB. It has downloaded from (https://wiki.csc.calpoly.edu/datasets/wiki/apriori)
2. Chess: the database originally generated and described by Alen Shapiro. The data from game field. It has 3196 games. And it is contain 36 attribute. The attributes values are categorize. And the data has class, so it is suitable for classification mining. It has been downloaded from (http://fimi.ua.ac.be/data/)
3. Mushroom: This data set includes descriptions of hypothetical samples corresponding to 23 species of gilled mushrooms. Each species is identified as definitely edible, definitely poisonous, or of unknown edibility and not recommended. This latter class was combined with the poisonous one. It is has 23 attributes one of them is class. Also it is has 8124 Instances (transactions). the data set exists here (http://fimi.ua.ac.be/data/)
4. T10|4D100K Dataset: The dataset T10|4D100k, it is a real dataset, it is represent market basket, and it was obtained from http://fimi.ua.ac.be/data/, contains a hundred thousand transactions. This dataset contains an item from an itemset of 1000 items.

### Statistic evaluation

In this subsection, the statistic calculation has been done on many datasets. In order to study the transaction lengths of the datasets. It is shown that the assumption, trepidation for the size of the itemset is 2^*n*^ [4], where n is the total number of the set of items. In this subsection, the focus is on checking whether the assumption 2^*n*^ is logical or not.

**Fig 8:**
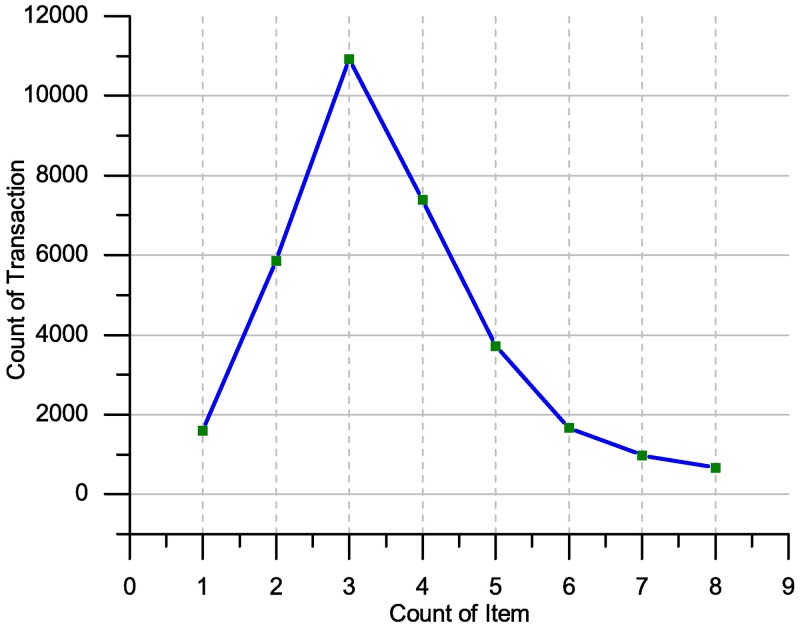
Item count representation on Apriori dataset 75K.T.

We analyzed the **Apriori dataset**. Based on the above assumption probability, the itemset length is up to 49 items. In each transaction, the itemset list counts up to 5.6*10^14^. In Fig 8, we show the tally of the items (in the form of a graph) occurring during the transactions. The vast majority of length transactions are between 1 and 6 items. From (Fig 8), the length of the transactions and items occurrence shows that almost 1588 transactions have only one item. In contrast, 5849 transactions have two items, 10914 transactions have three items, 7399 transactions have four items, 3720 transactions have five items, 1666 transactions have six items, 974 transactions have seven items and 657 transactions have eight items. These results show clearly that more than third of the transactions has only three items. Moreover, the maximum length of the transaction is equal to eight items. Thus, the ITDApriori is able to produce all the itemsets probability that have a length between 2 and 8 which is close to 45*10^7^ itemsets. This covers all plausible transactions close to the itemset only. Through experience, we found only 396 itemsets with minimum support of 0.00133%. In other words, all itemsets occurred at least once. The itemset count is only 396 and covers all the itemsets that occurred in the dataset. This number cannot be compared with 45*10^7^ because of the large difference.

According to the **chess dataset**. It has 76-possibility value, but is arrange in categorized fields. So, it has fixed attributes length, it is 36 attributes. If we calculate, the itemset upon to the rule 2^76^ is equal to 7.5*10^22^. But the actual length of the dataset, for all possibility even those itemsets did not exist in the dataset, is 6*10^10^. Based on experiment in the chess dataset, the actual length of itemsets is almost six thousands elements in ITDM.

When the **Mushroom dataset** has been studied, it has found it has 119 elements, those are categorized in 23 fields. The possibility of itemset length has calculated, based on the assumption 2^*n*^ (2^119^), it is equal to 6*10^35^. But the actual length of the dataset, for all possibility, is 8*10^13^. The experiment finds the actual itemset length is almost two thousands elements.

**Fig 9:**
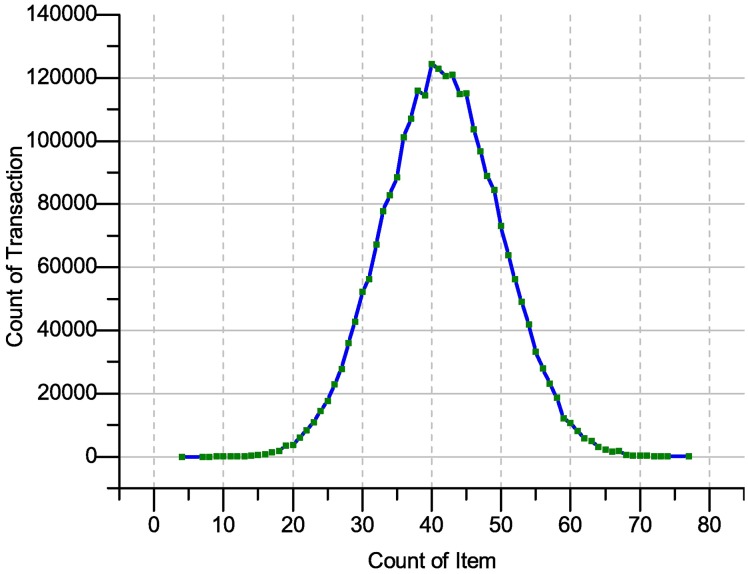
Transaction length representation on (T10|4D100K) database.

The representative of the item in the transaction, of **T10|4D100k dataset**, is shown in Fig 9. The x-axis is the size of the transaction, and the y-axis the number of transactions. According to 2^*n*^ assumption, the itemset is equal to 2^1000^, indicating that the size of the itemset is up to 1*10^301^. However, the transaction with the maximum number of items in this dataset has only 29 items. This means that the number of possible itemsets cannot exceed a total of 3*10^29^ million. Based on the statistical results, only 3*10^5^ itemsets have a support rate greater than 0.001%, indicating that each itemset in the transaction occurs at least once.

**Fig 10:**
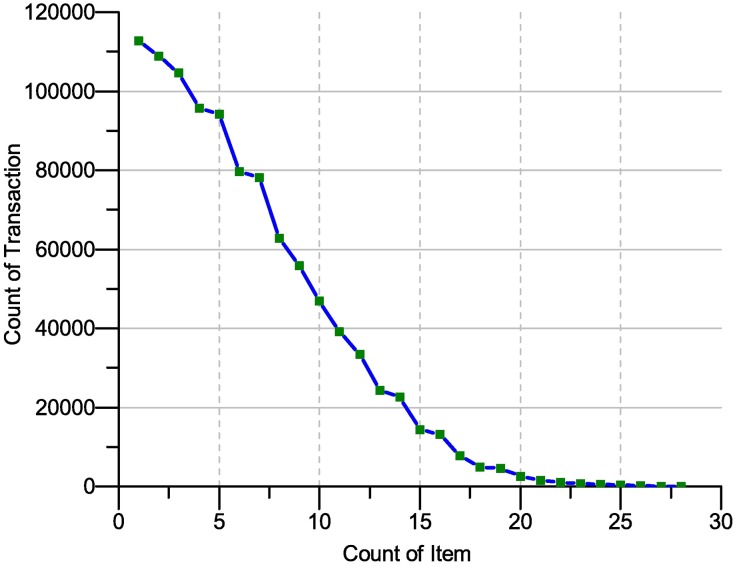
Item count representation on (T10|4D100K) database.

Fig 10 shows a representation of the items in the sample dataset. As shown in the diagram, more than 50% of the items occurred in less than a thousand transactions. The x-axis represents the items, and the y-axis represents the occurrence of these items in the database transaction. From the Fig 10, More than 50% of the items have support less than 1%. The above result for the dataset sample means that if the clients state the support to be less than or equal to 1%, more than 50% of the items will be discarded from the frequent itemset. Moreover, more than 25% of the items are covered in the dataset and less than 0.05% in the database transaction. Hence, the researcher must cover this number of items. The next sub-section discus the scalability briefly.

### Results on executing time for counting support

**Fig 11:**
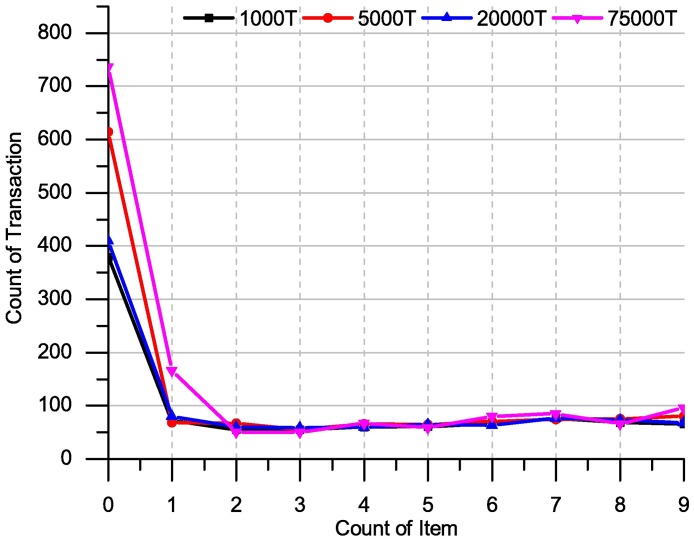
Execution times over many support, and different size of the Apriori dataset for ITD Apriori algorithm.

This sub-section we present results on executing time for counting support on different size of data. The experiments are run in C# programming language with computer specifications 2 Duo CPU (Intel E4500) 2.2 GHZ, with RAM 4 GB, under windows 7. Fig 11 shows how ITDApriori scales up as the number of transactions is increased from one thousand to 75 thousands transactions.

**Fig 12:**
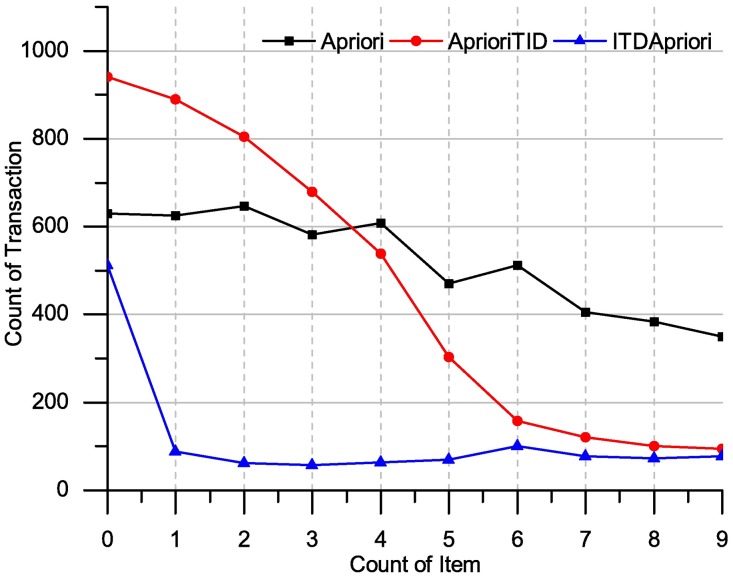
Execution times over many support: Apriori, TDApriori and ITDApriori on chess dataset.

From Fig 11, we used the Apriori dataset [31] with different size 1K, 5K, 20K and 75K for the average sizes of transactions. Tens of experiments have done and an average of execution time calculated according to minimal support, where the value minimum support was between 0% and 10%. The execution times are normalized in Fig 12. Proximately, the execution time after first mining is equal between all sizes of the dataset. Just the first mining consumes little bit time (710 Millisecond less than one second) where the volume of is up to 75K transactions, where the size and volume of the database are a big challenge for the most of the mining technique (as the execution time result shown that the ITDApriori can carry big database without consuming a lot of time).

The other dataset: chess, mushroom and T10|4D100K datasets have only one database file. Thus, the scalability is discuss over the Apriori dataset, where it has four database files. However, the scalability of ITD Apriori was tacit in the next sub-section where it is comparison the ITD Apriori with other algorithms over set of dataset.

### Comparison results

**Fig 13:**
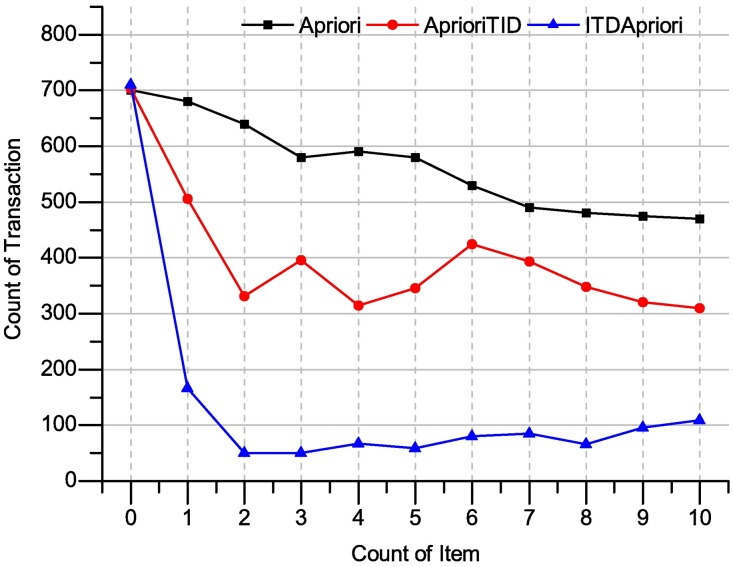
Execution times over many support: Apriori, AprioriTID and ITDApriori on Apriori dataset.

The ITDApriori together-with Apriori, and AprioriTID are applied over the benchmark transaction dataset Apriori. The execution time of many support values ranging from 0–10 over the above mentioned datasets has been presented in Figs 12 and 13 on the dataset chess and Apriori dataset respectively.

The ITD Apriori, ApioriTID and Apriori has applied over the chess dataset and the result shows ITDApriori outperforms. In the first step, when ITD Apriori generates ITDM Itemset, it consumes little bit of time compared to other two algorithms. Onwards extractions, for instance, when 1% support applied as shown in Fig 12 gives the best results.

In the following table, we present the improvement of ITDApriori to Apriori, TDApriori in term of execution time.

From Table 23, the algorithm ITD Apriori has been applied to the chess dataset. The execution time over this dataset for proposed algorithm is best compared to other algorithms. For instance, in the third line (where support = 2), the Apriori algorithm requires 647 ms, AprioriTID requires 804 ms and ITD Apriori needs only 61.6 ms. In other word, the proposed method has improvement more than 1000% and this is magic touch.

**Table 23:** The executing time (in Millisecond) comparison among Apriori, TDApriori and ITDApriori on chess data.

Support	Apriori	AprioriTID	Proposed ITDApriori	Improvement (%)
0	630	940	512	53.32
1	625	890	88	760.80
2	647	804	62	1070.16
3	582	679	58	987.07
4	608	538	64	795.31
5	471	303	70	452.86
6	512	158	100	235.00
7	405	121	78	237.18
8	384	100	72	236.11
9	350	95	78	185.26
10	267	90	85	110.00

The Fig 13 clearly shows the promising results of the ITDApriori. In the first step when ITDM generates Intermediate Itemset, so it consumes little bit of time compared to the other two algorithms. Onwards extractions, for instance, when 1% support applied as shown in the figure archives the best results. In this case: many instances of frequent itemsets could be extracted without going back to the database.

In the following table, we present the improvement of ITDApriori to Apriori, AprioriTID in term of execution time.

From Table 24, the execution time has been reduced to two-third of the total time. The value of time represents for Apriori and TDApriori full mining, where the algorithms go to rebuild knowledge from the beginning every time. But, for ITD Apriori extracts knowledge from ITDM list. In this table, the result in row 0 (support = 0) 710 for ITD Apriori doesn’t derive frequent itemset set from ITDM only, but it also rebuilt the whole ITDM from the begging. In this case, ITD Apriori has been run without previous result, which means it going to create ITDM. However, the proposed algorithm make Improvement 1.2%. In the second line in Table 23 shows the reduced execution time, where Apriori requires 680 milliseconds (ms), AprioriTID requires 506 ms and ITD Apriori needs only 166 ms, that mean it improve the process 257.23%.

**Table 24:** The executing time (in Millisecond) comparison among Apriori, TDApriori and ITDApriori on apriori data.

Support	Apriori	TDApriori	Proposed ITDApriori	Improvement (%)
0	700	703	710	1.20
1	680	506	166	257.23
2	640	331	50	871.00
3	580	396	50	876.00
4	590	314	67	574.63
5	580	346	59	684.75
6	530	425	80	496.88
7	490	393	85	419.41
8	480	348	66	527.27
9	475	320	96	314.06
10	470	310	109	257.80

**Fig 14:**
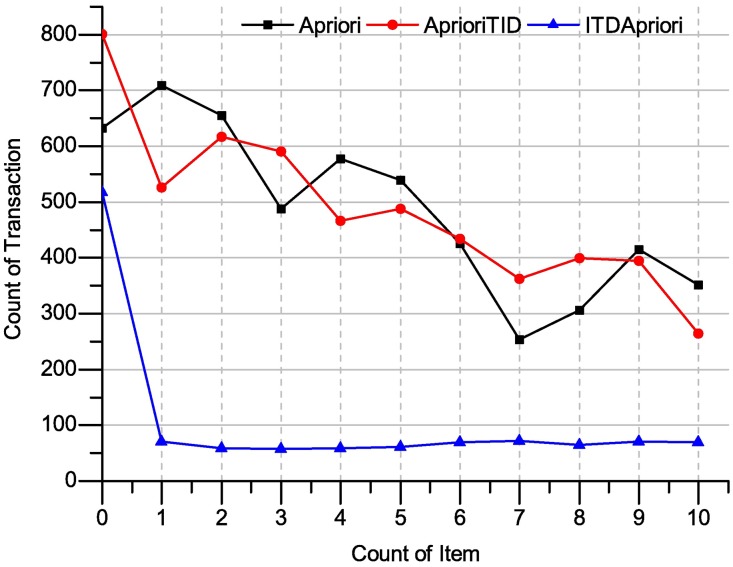
Execution times over many support: Apriori, TDApriori and ITDApriori on mushroom dataset.

In the Mushroom dataset, Apriori, AprioriTID and TID Apriori have applied, and the execution time result has presented in the Fig 14. As the Fig 14 shows that ITD Apriori has outperforms.

In the following table, we present the improvement of ITDApriori to Apriori, TDApriori in term of execution time over mushroom dataset.

From Table 25, the three algorithms have been applied to the mushroom dataset. The execution time over this dataset is appears in the Table. The best result is for ITD Apriori algorithm. For instance, in the sixth line (where support = 5), the Apriori algorithm requires 539.09 ms, AprioriTID requires 487.87 ms and ITD Apriori needs only 58.6 ms. In this case, the ITD Apriori make improvement 741.80% and this make mine easy and at no time.

**Table 25:** The executing time (in Millisecond) comparison among Apriori, TDApriori and ITDApriori on mushroom dataset.

Support	Apriori	AprioriTID	Proposed ITDApriori	Improvement (%)
0	632	801	518	38.32
1	709	526	70	782.14
2	655	617	58	996.55
3	488	591	57	846.49
4	578	466	59	784.75
5	539	488	61	741.80
6	426	434	69	523.19
7	253	362	72	327.08
8	306	399	65	442.31
9	415	395	70	478.57
10	351	264	69	345.65

**Fig 15:**
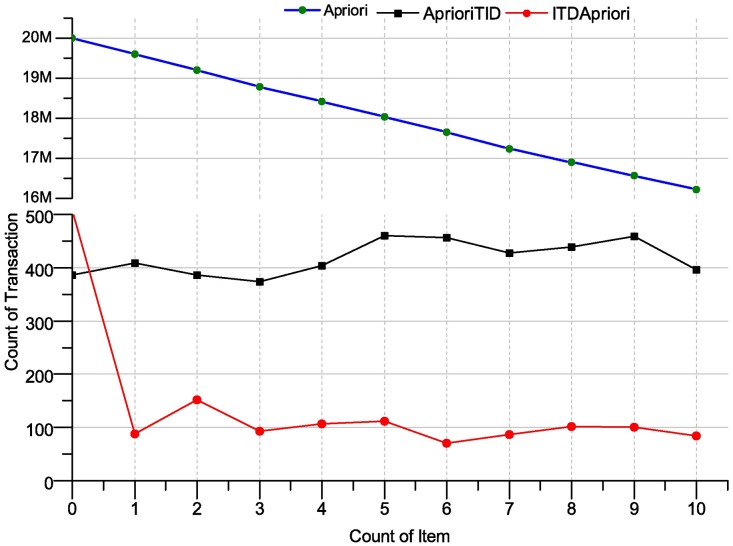
Execution times over many support: Apriori, AprioriTID and ITDApriori on T10|4D100K dataset.

When the set of algorithms (Apriori, AprioriTID and ITD Apriori) applied over the T10|4D100K dataset, on the machine with specifications appear in front of the previous section, we get a result on the AprioriTID and ITD Apriori, but for Apriori, we wait a lot of time without having any result. However, the result of two algorithms AprioriTID and ITD Apriori have been presented in Fig 15. The Fig 15 shows promising execution time of ITD Apriori. In the following table, we present the improvement of ITDApriori to Apriori, AprioriTID in term of executing time on T10|4D100K dataset.

The set of algorithms has been applied to the chess dataset. The execution time over this dataset has been presented in Table 26. The Apriori algorithm did not work over this dataset on experimental machine. The ITD Apriori is better than AprioriTID where the ITD Apriori save almost three-quarters of the time. For example, in the last row (where support = 10), Apriori needs more than five hour, AprioriTID requires 395.9 ms and ITD Apriori needs only 84.4 ms. The ITD Apriori is make a lot of improvement over two other algorithms, if you calculate the improvement between AprioriTID and ITD Apriori, also, it is making a good improvement.

**Table 26:** The executing time (in Millisecond) comparison among Apriori, TDApriori and ITDApriori on T10|4D100K dataset.

support	Apriori	AprioriTID	Proposed ITDApriori	Improvement (%)
0	20000000	386	509	1964574
1	19604746	408	87	11267230
2	19205401	386	151	6359432
3	18785613	374	92	10209676
4	18423390	404	107	8609150
5	18031557	460	111	8122430
6	17654125	456	70	12610315
7	17240452	427	87	9908451
8	16901257	438	102	8285045
9	16571963	458	100	8286111
10	16212692	396	84	9650548

## Conclusion and future remarks

The paper presents an ARM approach where a new itemset format structure is adopted to address the problem of threshold that necessitates rescanning the entire database. Our novel approach, on the other hand, prepares knowledge or a frequent itemset with all possible itemsets occurring in the database as an intermediate step to obtain the final instance of the frequent itemset. Moreover, the approach creates an intermediate itemset and applies a threshold to extract categorical frequent itemsets with diverse threshold values. Thus, improving the overall efficiency as we no longer need the algorithm to rescan the entire database. The proposed algorithm also helps to extract many frequent itemsets according to a pre-determined minimum support with an independent purpose. Furthermore, the association rule set is extracted with high confidence and weak support. Additionally, the proposed approach is capable to be deployed in any mining system in a fully parallel mode; consequently, increasing the efficiency of the association rules discovery process and making it feasible for real-time applications. Finally, that paper presents that the proposed approach outperforms the extant state-of-the-art and shows promising results.

In future, we are willing to apply our method on other data mining techniques such as classification and clustering. This motivation is because the main stage of classification and clustering requires finding frequent pattern related to the specific classes or clusters. Moreover, we are also willing to generalize the proposed algorithm to handle the incremental learning problem, which one of the most desirable ARM open issues.
