# Correction: A novel association rule mining approach using TID intermediate itemset

**DOI:** 10.1371/journal.pone.0196957

**Published:** 2018-05-01

**Authors:** Iyad Aqra, Tutut Herawan, Norjihan Abdul Ghani, Adnan Akhunzada, Akhtar Ali, Ramdan Bin Razali, Manzoor Ilahi, Kim-Kwang Raymond Choo

There is an error in affiliation 1 for authors Iyad Aqra, Tutut Herawan, and Norjihan Abdul Ghani. Affiliation 1 should be: Department of Information Systems, Faculty of Computer Science and Information Technology, University of Malaya, Kuala Lumpur, Malaysia.

The following information is missing from the Funding section: This research is supported by University of Malaya Postgraduate Research Grant (PPP) no. vote PG106-2015B, and it is supported by Faculty of Computer Science & Information Technology University of Malaya.
